# Association and Interaction Effects of Interleukin-12 Related Genes and Physical Activity on Cognitive Aging in Old Adults in the Taiwanese Population

**DOI:** 10.3389/fneur.2019.01065

**Published:** 2019-10-09

**Authors:** Eugene Lin, Po-Hsiu Kuo, Yu-Li Liu, Albert C. Yang, Shih-Jen Tsai

**Affiliations:** ^1^Department of Biostatistics, University of Washington, Seattle, WA, United States; ^2^Department of Electrical & Computer Engineering, University of Washington, Seattle, WA, United States; ^3^Graduate Institute of Biomedical Sciences, China Medical University, Taichung, Taiwan; ^4^Department of Public Health, Institute of Epidemiology and Preventive Medicine, National Taiwan University, Taipei, Taiwan; ^5^Center for Neuropsychiatric Research, National Health Research Institutes, Hsinchu, Taiwan; ^6^Division of Interdisciplinary Medicine and Biotechnology, Beth Israel Deaconess Medical Center/Harvard Medical School, Boston, MA, United States; ^7^Institute of Brain Science, National Yang-Ming University, Taipei, Taiwan; ^8^Department of Psychiatry, Taipei Veterans General Hospital, Taipei, Taiwan; ^9^Division of Psychiatry, National Yang-Ming University, Taipei, Taiwan

**Keywords:** Alzheimer's diseases, cognitive aging, cognitive impairment, interleukin-12, neurodegeneration

## Abstract

Evidence suggests that the neuro-inflammation mechanisms associated with interleukin-12 (IL-12) may be linked to Alzheimer's diseases and cognitive aging. In this study, we speculate that single nucleotide polymorphisms (SNPs) in IL-12-associated genes, such as *IL12A, IL12B, IL12RB1*, and *IL12RB2* genes, could be associated with cognitive aging individually and/or via complicated interactions in the elder Taiwanese population. There were totally 3,730 Taiwanese individuals with age ≥60 years from the Taiwan Biobank. Mini-Mental State Examination (MMSE) was analyzed for all participants. We employed MMSE scores to assess cognitive functions. Our analysis revealed that the *IL12A* gene (including rs116910715, rs78902931, and rs78569420), the *IL12B* gene (including rs730691), and the *IL12RB2* gene (including rs3790558, rs4655538, rs75699623, and rs1874396) were associated with cognitive aging. Among these SNPs, the association with the *IL12RB2* rs3790558 SNP remained significant after performing Bonferroni correction (*P* = 6.87 × 10^−4^). Additionally, we found that interactions between the *IL12A* and *IL12RB2* genes influenced cognitive aging (*P* = 0.022). Finally, we pinpointed the effects of interactions between *IL12A, IL12B*, and *IL12RB2* with physical activity (*P* < 0.001, = 0.002, and < 0.001, respectively). Our study suggests that the IL-12-associated genes may contribute to susceptibility to cognitive aging independently as well as through gene-gene and gene-physical activity interactions.

## Introduction

Interleukin-12 (IL-12) is a pro-inflammatory cytokine that builds a key link between adaptive immunity and innate resistance (1). IL-12 is a heterodimer composed of the IL-12α (also known as IL-12p35) and IL-12β (also known as IL-12p40) (1). Furthermore, IL-12 binds to the IL-12 receptor, which is a heterodimeric receptor formed by the IL-12R-β1 and IL-12R-β2 subunits (1). There is growing evidence that IL-12 is regulated in neuro-inflammatory processes associated with neurodegenerative disorders such as Alzheimer's disease (AD) and mild cognitive impairment (2, 3). It has been observed that IL-12 contributes to cognitive decline such as reduced performance in processing speed for elderly individuals aged 70–90 years in an Australia population (4). In the same cohort, a subtype of mild cognitive impairment (that is, non-amnestic multiple domain) was associated with higher levels of IL-12 and IL-12β (5). Moreover, a meta-analysis study revealed that there were significantly higher levels of IL-12 in AD patients when compared with healthy controls in peripheral blood (6). In addition, a recent study simultaneously assessed 242 blood proteins in 80 older adults with remitted major depression and found IL-12β to serve as one of the three proteins to predict cognitive impairment by using a machine learning prediction model (7). It has also been suggested that IL-12β is involved in affecting Mini-Mental State Examination (MMSE) test scores and gray-matter volumes of lateral prefrontal cortex and hippocampus in older adults (8). Furthermore, it has been reported that elderly persons with inadequate physical activity showed higher levels of IL-12β, smaller gray-matter volumes, and more cognitive decline than active elderly persons, suggesting probable gene-physical activity interactions (8).

Among the genes related to IL-12 are the interleukin 12A (*IL12A*), interleukin 12B (*IL12B*), interleukin 12 receptor subunit beta 1 (*IL12RB1*), and interleukin 12 receptor subunit beta 2 (*IL12RB2*) genes. The *IL12A* gene is located on chromosome 3q25.33 and encodes the IL-12α subunit (1). The *IL12B* gene is located on chromosome 5q33.3 and encodes the IL-12β subunit (1). It has been demonstrated that single nucleotide polymorphisms (SNPs) in *IL12A* (including rs568408) and *IL12B* (including rs3212227) genes were likely to influence late-onset AD in a Chinese population (9). In addition, Enright et al. (10) reported that the *Il12b* knockout male mice exhibited a significant increase in the average time to reach the platform in the Morris water navigation task (also known as the Morris water maze; a test of spatial learning for rodents), when compared to the wild-type. In the APP/PS1 mouse model of AD, Vom Berg et al. (11) also found an increased production of the common IL-12 and IL-12β subunit in microglia, the primary immune cells of the central nervous system. Furthermore, Vom Berg et al. (11) demonstrated that the genetic ablation of the *Il12b* gene or peripheral administration of a neutralizing IL-12β-specific antibody may contribute to a decreased cerebral amyloid load in APP/PS1 mice.

The *IL12RB1* gene is located on chromosome 19p13.11 and encodes the IL-12R-β1 subunit (1). Furthermore, the *IL12RB2* gene is located on chromosome 1p31.3 and encodes the IL-12R-β2 subunit (1). The *IL12RB1* and *IL12RB2* genes are thought to contribute to the host defense and inflammatory response (1). de Beaucoudrey et al. reported that loss-of-function mutations in the *IL12RB1* gene may debilitate the development of human IL-17-producing T cells in an *in vivo* study, where IL-17 has been implicated in the pathogenesis of AD-related neuroinflammation (12–14). Additionally, several SNPs (such as rs12119179, rs1495965, and rs924080) near the *IL12RB2* gene were found to be associated with Behcet's disease in genome-wide association studies, were the disease can lead to inflammation in the brain and central nervous system (15, 16).

In reference to the aforementioned considerations, it was hypothesized that IL-12 associated genes may play a significant role in the pathogenesis of age-dependent cognitive decline and the development of cognitive aging. Therefore, we presumed that IL-12 relevant genes, namely the *IL12A, IL12B, IL12RB1*, and *IL12RB2* genes, might be associated with cognitive aging. To the best of our knowledge, the effects of these IL-12 relevant genes on cognitive aging are limited with respect to human datasets. Thus, we investigated the interplays between cognitive aging and SNPs in the *IL12A, IL12B, IL12RB1*, and *IL12RB2* genes in the present association study. We also gauged the probable gene-gene and gene-physical activity interactions on cognitive aging.

## Materials and Methods

### Study Population

This study incorporated Taiwanese participants from the Taiwan Biobank, which collected specimens and relevant information from individuals in recruitment centers across Taiwan (17–22). Our study cohort was comprised of 3,730 subjects. There were the following two inclusion criteria: (1) individuals whose age were 60 years or over; and (2) individuals who were self-reported as being of Taiwanese Han Chinese ancestry (22). We excluded individuals with a history of cancer (22). Ethical approval for the study was granted by the Institutional Review Board of the Taiwan Biobank before performing the study (approval number: 201506095RINC). The approved informed consent form was signed by each subject. All experiments were achieved by means of proper regulations and guidelines.

We defined education according to whether or not high school was attended (20, 21). The definition of physical activity was the measurement of exercise activities at least three times in a week and at least 30 min each time (20, 21).

### Cognitive Assessment

We performed global cognitive assessment by using the 30-point MMSE, which encompasses questions according to the five areas of recall, registration, language, attention and calculation, and orientation (18). We evaluated MMSE both as a continuous phenotype and as a binary phenotype according to the following previously defined MMSE thresholds (23): MMSE score ≥24 (normal) and MMSE score < 24 (cognitive impairment). The cognitive assessment was conducted in the local languages (such as Taiwanese and/or Mandarin). The cognitive cut-off score of 24 was derived from previous studies (23) and was based on a Taiwanese version of MMSE.

### Genotyping

DNA was isolated from blood samples by employing QIAamp DNA blood kits following the manufacturer's instructions (Qiagen, Valencia, CA, USA). The quality of the isolated genomic DNA was carried out by utilizing agarose gel electrophoresis, and the quantity was completed by spectrophotometry (24). SNP genotyping was evaluated by employing the custom Taiwan BioBank chips, which were accomplished by using the Axiom Genome-Wide Array Plate System (Affymetrix, Santa Clara, CA, USA). The custom Taiwan BioBank chips were created to collect genetic profiles in Taiwanese subjects by utilizing SNPs on the Axiom Genome-Wide CHB 1 Array (Affymetrix, Santa Clara, CA, USA) with minor allele frequencies (MAFs) ≥5% and the Human Exome BeadChip (Illumina, Inc., San Diego, CA, USA) with MAFs > 10% (22). We searched for IL-12-associated variants by referring to the complete list of IL-12-associated genes/SNPs available in the custom Taiwan BioBank chips. The SNP panel consisted of 75 SNPs from the following four genes, namely the *IL12A, IL12B, IL12RB1*, and *IL12RB2* genes (Supplementary Table 1). In addition, we performed quality control procedures for excluding SNPs from subsequent analysis as follows (25). Nine SNPs were excluded because of being unable to achieve Hardy-Weinberg equilibrium (with a *P*-value < 0.05) or because of a genotyping call rate <95%. Supplementary Table 1 shows the genotyping results, including genotyping call rates, *P*-values for Hardy-Weinberg equilibrium, and MAFs. Additionally, we filtered SNPs and then selected 35 tag SNPs by using PLINK (26) with a linkage disequilibrium value of *r*^2^ = 0.8 as a threshold (Supplementary Table 2).

### Statistical Analysis

In this study, linear regression analysis was conducted to assess the relationship between MMSE scores and our variables of interest such as age, gender, and education. In addition, we determined the association of the investigated SNP with MMSE scores by a general linear model using age, gender, and education as covariates (27). The genotype frequencies were weighed for Hardy-Weinberg equilibrium to detect genotyping errors (28) by utilizing a χ^2^ goodness-of-fit test with one degree of freedom (that is, the number of genotypes minus the number of alleles). Adjustments for multiple testing were performed by using the Bonferroni correction. The criterion for significance was defined as *P* < 0.05 for all tests. Data were shown by the mean ± standard deviation.

In order to explore gene-gene and gene-physical activity interactions, we used the generalized multifactor dimensionality reduction (GMDR) method (29). We analyzed two-way interactions by utilizing 10-fold cross-validation. The GMDR method generated several output parameters, such as empirical *P*-values and the testing accuracy, to estimate each chosen interaction. Furthermore, covariates such as age, gender, and education were provided for gene-gene and gene-physical activity interaction analysis in our interaction models. We completed the empirical *P*-value of the testing accuracy for each chosen interaction by using permutation testing (based on 1,000 shuffles).

In this study, there were missing data in the genotypic data (as shown in Supplementary Table 1) and no missing data in the phenotypic data. In GMDR, a missing genotype is imputed proportional to the frequencies of the SNPs observed at this locus, and insignificant missing data will not affect the analysis (29). Because the selected SNPs possess <0.5% missing genotypic data by excluding SNPs with a genotyping call rate <95% (Supplementary Table 1), we have chosen to use GMDR after assessing the influence of missing data. In order to correct for multiple testing, we employed the Bonferroni correction.

## Results

### Study Cohort

Table 1 illustrates the clinical and demographic characteristics of our study cohort, which consisted of 3,730 individuals. The median MMSE score was 28 and the interquartile range was 26–29. In this study, we found that correlations between MMSE score with age (*P* = 8.04 × 10^−10^), gender (*P* = 1.83 × 10^−7^), and education (*P* = 2.2 × 10^−16^) were significant.

**Table 1:** Demographic and clinical characteristics of study subjects.

**Characteristic**	**Overall**
No. of subjects, *n*	3,730
Mean age ± SD, years	64.8 ± 3.5
Female, %	50.27
Less than high school graduate, *n*	1,735
Physical activity, *n*	2,427
MMSE score, median (IQR)	28 (26–29)

*IQR, interquartile range; MMSE, Mini-Mental State Examination; SD, standard deviation. Data are presented as mean ± standard deviation*.

### Association of Cognitive Aging in *IL12A, IL12B*, and *IL12RB2*

First, we explored the associations between cognitive aging and four IL-12 related genes, namely the *IL12A, IL12B, IL12RB1*, and *IL12RB2* genes. Among the 35 tag SNPs investigated in the present study (Supplementary Table 2), there were 8 tag SNPs within the *IL12A, IL12B*, and *IL12RB2* genes revealing evidence of associations (*P* < 0.05) with MMSE scores (Table 2). These 8 tag SNPs were the rs116910715 (recessive model: *P* = 0.0031), rs78902931 (recessive model: *P* = 0.0388), and rs78569420 (recessive model: *P* = 0.0093) SNPs in the *IL12A* gene; the rs730691 SNP (dominant model: *P* = 0.0472) in the *IL12B* gene; and the rs3790558 (dominant model: *P* = 6.87 × 10^−4^), rs4655538 (dominant model: *P* = 0.0454), rs75699623 (dominant model: *P* = 0.0308), and rs1874396 (dominant model: *P* = 0.0425) SNPs in the *IL12RB2* gene. In this study, we only found the genotyping data for the rs7412 SNP, but not for the rs429358 SNP in the *APOE* gene. Based on the rs7412 SNP, the frequency of the *APOE*-ε2 allele in patients showing normal cognition vs. cognitive impairment was 14.2 vs. 18.9%. However, we were unable to assess *APOE*-ε4 carrier status.

**Table 2:** Linear regression models of associations between the MMSE scores and 8 tag SNPs within the *IL12A, IL12B*, and *IL12RB2* genes, which have an evidence of association (*P* < 0.05).

						**Dominant model**			**Recessive model**		
**Gene**	**CHR**	**SNP**	**A1**	**A2**	**MAF**	**BETA**	**SE**	***P***	**BETA**	**SE**	***P***
*IL12A*	3	rs116910715	A	G	0.065	−0.14	0.13	0.3012	−1.99	0.67	0.0031
		rs78902931	G	A	0.128	−0.03	0.10	0.7782	−0.77	0.37	0.0388
		rs78569420	T	G	0.083	−0.06	0.12	0.6042	−1.26	0.48	0.0093
*IL12B*	5	rs730691	T	C	0.440	−0.19	0.09	0.0472	−0.03	0.11	0.8141
*IL12RB2*	1	rs3790558	G	T	0.468	−0.33	0.10	**6.87 × 10^−4^**	−0.17	0.11	0.1186
		rs4655538	T	C	0.186	−0.19	0.09	0.0454	−0.01	0.24	0.9679
		rs75699623	A	G	0.069	0.28	0.13	0.0308	−0.10	0.59	0.8652
		rs1874396	G	T	0.227	−0.18	0.09	0.0425	0.00	0.19	0.9891

*A1, minor allele; A2, major allele; BETA, Beta coefficients; Chr, chromosome; HWE, Hardy–Weinberg equilibrium; MAF, minor allele frequency; MMSE, Mini-Mental State Examination; SE, standard error. Analysis was obtained after adjustment for covariates including age, gender, and education. P-values which remain significant after performing Bonferroni correction are shown in bold*.

### Association Between Cognitive Aging and *IL12RB2* rs3790558

Moreover, as illustrated in Table 2, the significance persisted for the association with MMSE scores after employing Bonferroni correction [*P* < 0.05/(35 × 2) = 7.14 × 10^−4^] for the rs3790558 SNP (dominant model: *P* = 6.87 × 10^−4^) in the *IL12RB2* gene.

### Gene-Gene Interaction Analysis

Next, we utilized categorized MMSE scores as an outcome (normal: MMSE score ≥24; cognitive impairment: MMSE score < 24) for gene-gene interaction analysis. The GMDR method was employed to estimate the effects of consolidation among the 8 tag SNPs in cognitive aging, incorporating age, gender, and education as covariates. Table 3 describes the results generated from the GMDR method for two-way gene-gene interaction analysis using covariate adjustment. As illustrated in Table 3, there was a significant two-way model concerning *IL12RB2* rs4655538 and *IL12A* rs78902931 (*P* = 0.022), suggesting a probable gene-gene interaction between *IL12RB2* rs4655538 and *IL12A* rs78902931 in regulating cognitive aging. Likewise, there was a significant two-way gene-gene interaction model concerning *IL12RB2* rs4655538 and *IL12A* rs78569420 (*P* = 0.036) in regulating cognitive aging. In addition, there was a significant two-way SNP-SNP interaction model concerning *IL12RB2* rs3790558 and *IL12RB2* rs4655538 (*P* = 0.017) in influencing cognitive aging. However, the effect of these gene-gene and SNP-SNP interaction models did not remain significant after Bonferroni correction.

**Table 3:** Gene-gene interaction models identified by the GMDR method with adjustment for age, gender, and education.

**2-way interaction model**	**Testing accuracy (%)**	***P*-value**
*IL12RB2* rs4655538, *IL12A* rs78902931	53.43	0.022
*IL12RB2* rs4655538, *IL12A* rs78569420	53.17	0.036
*IL12RB2* rs3790558, *IL12RB2* rs4655538	53.87	0.017

*GMDR, generalized multifactor dimensionality reduction. P-value was based on 1,000 permutations. Analysis was obtained after adjustment for covariates including age, gender, and education*.

### Physical Activity and Gene Interaction Analysis

Table 4 illustrates the GMDR method of physical activity and gene interaction analysis in cognitive aging by utilizing age, gender, and education as covariates. There were significant two-way models concerning *IL12A* (including rs116910715, rs78902931, and rs78569420) and physical activity (*P* = 0.002, < 0.001, and 0.001, respectively), indicating potential physical activity and gene interactions between *IL12A* and physical activity in regulating cognitive aging. Likewise, there was a significant two-way model concerning *IL12B* rs730691 and physical activity (*P* = 0.002). Finally, there were significant two-way models concerning *IL12RB2* (including rs3790558, rs4655538, s75699623, and rs1874396) and physical activity (*P* = 0.003, < 0.001, < 0.001, and 0.025, respectively). The effect of these physical activity and gene interaction models remained significant after Bonferroni correction (*P* < 0.05/8 = 0.006) except the interaction model between physical activity and *IL12RB2* rs1874396.

**Table 4:** Physical activity and gene interaction models identified by the GMDR method with adjustment for age, gender, and education.

**2-way interaction model**	**Testing accuracy (%)**	***P*-value**
Physical activity, *IL12A* rs116910715	54.94	**0.002**
Physical activity, *IL12A* rs78902931	55.66	**<0.001**
Physical activity, *IL12A* rs78569420	54.81	**0.001**
Physical activity, *IL12B* rs730691	54.82	**0.002**
Physical activity, *IL12RB2* rs3790558	54.82	**0.003**
Physical activity, *IL12RB2* rs4655538	55.90	**<0.001**
Physical activity, *IL12RB2* rs75699623	54.32	**<0.001**
Physical activity, *IL12RB2* rs1874396	53.41	0.025

*GMDR, generalized multifactor dimensionality reduction. P-value was based on 1,000 permutations. Analysis was obtained after adjustment for covariates including age, gender, and education. P-values of <0.006 (Bonferroni correction: 0.05/8) are shown in bold*.

## Discussion

The present study is the first to date to identify whether the major impacts of 35 tag SNPs within four IL-12-associated genes, namely the *IL12A, IL12B, IL12RB1*, and *IL12RB2* genes, are significantly linked to the risk of cognitive aging individually and via gene-gene and gene-physical activity interactions in elder Taiwanese subjects. Here, we reveal for the first time that the *IL12RB2* gene might play a vital role in modulating cognitive aging in elder Taiwanese individuals. Intriguingly, the significant persisted for the association of the key rs3790558 SNP in the *IL12RB2* gene with MMSE scores after correcting for multiple testing (*P* < 7.14 × 10^−4^). The rs3790558 SNP is located in the intron of *IL12RB2* gene. To investigate the possible roles of the rs3790558 SNP as expression quantitative trait locus, we employed HaploReg (http://compbio.mit.edu/HaploReg) to predict a possible functional role of this SNP. We found that the rs3790558 SNP is associated with the regulation of expressions in *IL12RB2* gene within transformed fibroblasts/cells and in blood tissues (30, 31). Additionally, our data revealed that gene-gene and gene-physical activity interactions between the IL-12-associated genes may contribute to the etiology of cognitive aging.

To the best of our knowledge, the present study is the first to raise the possibility that the key rs3790558 SNP in the *IL12RB2* gene might be associated with cognitive aging. Remarkably, the significant association between this key SNP and MMSE scores persisted even after applying Bonferroni correction. The functional relevance of *IL12RB2* rs3790558 on cognitive aging remains to be elucidated. To our knowledge, no other studies have been conducted to pinpoint *IL12RB2* rs3790558 with cognitive aging or age-related cognitive decline. Based on the aforementioned implications, we hypothesized that the IL-12 relevant genes such as *IL12RB2* might contribute to cognitive aging because IL-12 is implicated as a risk biomarker for AD and cognitive aging (1, 4, 7–9). Our findings further support previous animal studies, which demonstrated that inhibition of IL-12 signaling may reduce cognitive decline (11, 32). In addition, genome-wide association studies by Remmers et al. (16) and by Mizuk et al. (15) have identified an association of the *IL12RB2* gene with Behcet's disease at genome-wide significance. It has been shown that patients with Behcet's disease often suffer from irreversible loss of cognitive function in conjunction with various neurological disturbances in the central nervous system (33). In an Il12rb2 knockout mice study, Airoldi et al. (34) also reported that lack of Il12rb2 signaling may result in increased susceptibility to autoimmunity and immunopathology. Moreover, it has been suggested that the immune system and autoimmunity may play a role in the etiology of age-associated cognitive decline and AD (35–37). Furthermore, Li et al. (38) observed that the increased risks of dementia and AD in patients with autoimmune disorders. Interestingly, the *IL12RB2* rs3790558 SNP is a strong candidate for autoimmune disorders as this SNP has been previously implicated in autoimmune disorders such as systemic sclerosis. For example, Bossini-Castillo et al. (39) identified *IL12RB2* rs3790558 to be associated with systemic sclerosis, a disorder that is characterized by autoimmune dysfunction. It should be mentioned that the G allele frequency of *IL12RB2* rs3790558 varies considerably among different ethnic individuals, ranging from 46.8% in the present Taiwanese individuals, 43.7% in European individuals, 47.3% in East Asian individuals, 86.5% in African American individuals, to 48% in South Asian individuals as illustrated in public data from the 1000 Genomes Project (Supplementary Table 4).

Remarkably, we tracked down the interplay between the IL-12-associated genes (including *IL12A, IL12B*, and *IL12RB2*) and physical activity. This relationship might functionally manifest itself via epigenetic changes. Our finding is in agreement with other human and animal studies, indicating that physical activity may modulate inflammatory reactions through potential complex gene-physical activity interactions (8, 40, 41). In a previous population-based study of older adults, Papenberg et al. (8) reported that inactive older individuals may exhibit elevated levels of IL-12β, smaller gray-matter volumes, and poorer cognitive performance than older individuals with adequate physical activity.

On another note, our results also indicated the epistatic effects between the *IL12RB2* and *IL12A* genes in modulating cognitive aging by employing the GMDR method. To our knowledge, there are no previous findings available as no other studies have been investigated to assess gene-gene interactions among these genes. The biological effects of synergy between the IL-12-associated genes on cognitive aging remain to be elucidated.

A previous genetic association study found that *IL12A* rs568408 and *IL12B* rs3212227 SNP were significantly associated with late-onset AD risk (9). However, the present study showed no association of cognitive aging with these two SNPs (Supplementary Table 3). It is worth pointing out that various possible factors for the conflicting data include sample size, study design, covariate adjustment, phenotype definitions, and diverse ethnicities (21).

Among the strengths of our study is that we were able to utilize the Taiwan Biobank, the largest Taiwanese cohort, to conduct an extensive assessment of the IL-12-associated SNPs in cognitive aging (42, 43). In addition, we conducted the GMDR method to consider gene-gene interaction and gene-physical activity interaction in the model. However, because only the MMSE data were provided in the Taiwan Biobank, a major limitation is that a single measure of cognition (that is, MMSE) used as the cognitive assessment tool limits the rigor and depth of analytic inferences and associations with potential SNPs (44). Moreover, we did not control for other meaningful variables implicated in elderly cognitive decline (for example, insufficient characterization of the cohort including medical comorbidities and/or related medical burden) (44). Future studies are needed to develop a detailed assessment of the associations and interactions of probable SNPs with cognitive aging by leveraging specific cognitive domains (including executive, memory, language, and visuospatial function) in other worldwide populations (42, 44).

In conclusion, the present study completed a comprehensive investigation of the associations of cognitive aging with IL-12 relevant genes, namely the *IL12A, IL12B, IL12RB1*, and *IL12RB2* genes in old adults in the Taiwanese population. Moreover, the present study tested the impacts of gene-gene and gene-physical activity interactions among these genes in relation to cognitive aging. Mainly, if the current results are reproduced in statistically well-powered independent studies, the present study implicates the effects of the IL-12 relevant genes on the risk of cognitive aging individually and via complicated gene-gene and gene-physical activity interactions. This study pinpoints that IL-12 mediated signaling should be the focus of future studies on pathogenesis of age-dependent cognitive decline and a potential target for pharmacologic modulation. Independent studies with larger sample sizes will possibly establish further insights into the role of the IL-12 related genes suggested in this study.

## Data Availability Statement

The raw data supporting the conclusions of this manuscript will be made available by the authors, without undue reservation, to any qualified researcher.

## Ethics Statement

This study was approved by the Institutional Review Board of the Taiwan Biobank and complies with the Declaration of Helsinki. Informed written consent was obtained from all participants.

## Author Contributions

EL and S-JT: study conception and design. P-HK, Y-LL, and AY: acquisition of data. EL and S-JT: analysis and interpretation of data. EL: draft manuscript. All authors: read and approved the final manuscript.

### Conflict of Interest

The authors declare that the research was conducted in the absence of any commercial or financial relationships that could be construed as a potential conflict of interest.
