# Supplementary material for: Association and Interaction Effects of Interleukin-12 Related Genes and Physical Activity on Cognitive Aging in Old Adults in the Taiwanese Population
Source: Front Neurol. 2019 Oct 9;10:1065. doi: 10.3389/fneur.2019.01065 (PMC6795278; doi:10.3389/fneur.2019.01065)
Supplement: Supplementary file 1 [file Data_Sheet_1.pdf]

Supplementary information for

Association and interaction effects of interleukin-12 related genes and physical activity on cognitive aging in old adults in the Taiwanese population

Eugene Lin[1,2,3]*, Po-Hsiu Kuo[4], Yu-Li Liu[5], Albert C. Yang[6,7],

Shih-Jen Tsai[8,9]*

[1] Department of Biostatistics, University of Washington, Seattle, WA 98195, USA

[2] Department of Electrical & Computer Engineering, University of Washington, Seattle, WA 98195, USA

[3] Graduate Institute of Biomedical Sciences, China Medical University, Taichung, Taiwan

[4] Department of Public Health, Institute of Epidemiology and Preventive Medicine, National Taiwan University, Taipei, Taiwan

[5] Center for Neuropsychiatric Research, National Health Research Institutes, Miaoli County, Taiwan

[6] Division of Interdisciplinary Medicine and Biotechnology, Beth Israel Deaconess Medical Center/Harvard Medical School, Boston, MA 02215, USA

[7] Institute of Brain Science, National Yang-Ming University, Taipei, Taiwan

[8] Department of Psychiatry, Taipei Veterans General Hospital, Taipei, Taiwan

[9] Division of Psychiatry, National Yang-Ming University, Taipei, Taiwan

Supplementary Table 1. Genotyping results for 75 SNPs in four interleukin-12 related genes including the *IL12A*, *IL12B*, *IL12RB1*, and *IL12RB2* genes.

Gene	CHR	SNP	A1	A2	MAF	Genotyping call rate	P (HWE)
IL12A	3	rs11919494	T	C	0.096	0.998	0.419
		rs67717498	C	T	0.093	0.999	0.694
		rs12635350	A	G	0.192	0.999	0.631
		rs9864047	C	G	0.253	0.998	0.109
		rs72494860	G	A	0.151	**0.720**	0.614
		rs12631488	G	A	0.149	**0.272**	0.540
		rs7649651	A	C	0.398	0.999	0.743
		rs6441301	G	T	0.314	0.999	0.672
		rs6762955	A	T	0.057	0.998	0.646
		rs77444103	T	C	0.122	0.999	0.757
		rs9840016	C	T	0.274	0.997	0.182
		rs73154514	G	A	0.187	0.999	0.114
		rs11918072	C	T	0.174	0.999	0.236
		rs116910715	A	G	0.065	0.999	0.376
		rs6795437	C	T	0.203	0.999	0.201
		rs78902931	G	A	0.128	0.997	0.111
		rs7631387	G	A	0.177	0.999	0.171
		rs480350	G	A	0.226	0.999	0.477
		rs117988966	A	G	0.042	0.999	0.253
		rs1498736	A	T	0.191	0.999	0.707
		rs564799	T	C	0.167	0.999	**0.046**
		rs485789	T	G	0.166	0.998	0.052
		rs79458760	A	C	0.092	0.999	0.195
		rs67872958	C	T	0.192	0.999	0.612
		rs9880646	G	A	0.449	0.999	0.155
		rs74906756	T	C	0.097	0.998	0.476
		rs78569420	T	G	0.083	0.999	0.585

		rs4130283	G	A	0.265	0.998	**0.030**
		rs9853972	C	T	0.361	0.999	**0.017**
IL12B	5	rs3212227	G	T	0.442	0.996	0.866
		rs2853697	G	T	0.177	0.999	0.550
		rs2421047	A	G	0.441	0.998	0.749
		rs3213097	T	A	0.441	1.000	0.814
		rs3213094	T	C	0.455	0.997	0.515
		rs2569254	T	C	0.173	0.999	0.685
		rs3181218	T	C	0.455	0.997	0.482
		rs3181216	T	A	0.328	0.997	0.128
		rs3212220	A	C	0.455	0.998	0.462
		rs3212219	A	C	0.456	0.997	0.493
		rs3212217	G	C	0.456	1.000	0.582
		rs1003199	T	C	0.388	0.999	0.889
		rs2546893	G	A	0.492	0.999	0.895
		rs730690	A	G	0.440	0.998	0.545
		rs730691	T	C	0.440	0.995	0.195
		rs6894567	G	A	0.450	0.998	0.262
IL12RB1	19	rs445521	G	T	0.356	1.000	0.108
		rs383483	G	A	0.381	0.999	0.225
		rs372889	C	T	0.380	0.998	0.268
		rs3761041	T	C	0.309	1.000	0.095
		rs429774	C	T	0.361	0.999	0.156
		rs376008	T	C	0.358	0.999	0.087
		rs7250425	C	T	0.192	0.998	0.200
IL12RB2	1	rs7544381	T	C	0.173	0.999	0.794
		rs3790558	G	T	0.468	0.998	0.125
		rs2066445	T	C	0.225	0.998	0.122
		rs4655538	T	C	0.186	0.997	0.661
		rs2201584	A	G	0.324	0.998	0.955
		rs1495964	A	G	0.402	0.999	0.743
		rs17129794	C	A	0.058	0.999	0.324
		rs6693065	A	G	0.403	0.998	0.667

		rs4655699	T	C	0.346	0.996	0.463
		rs3790566	T	C	0.264	0.998	0.865
		rs3790567	A	G	0.256	**0.885**	**0.006**
		rs114967702	G	A	0.193	0.997	0.324
		rs3790569	C	T	0.389	0.999	0.972
		rs3828069	C	T	0.394	0.999	0.795
		rs881086	T	A	0.342	0.996	0.543
		rs79411372	T	C	0.073	1.000	0.903
		rs2228420	A	G	0.344	**0.228**	1.000
		rs2270614	G	A	0.341	0.999	0.645
		rs12133473	A	G	0.396	**0.228**	0.789
		rs6699474	A	G	0.397	0.999	0.890
		rs75699623	A	G	0.069	0.998	0.561
		rs2229546	A	C	0.375	**0.807**	0.313
		rs1874396	G	T	0.227	0.997	0.944

A1 = minor allele, A2 = major allele, Chr = chromosome, HWE = Hardy–Weinberg equilibrium, MAF = minor allele frequency, MMSE = Mini-Mental State Examination.

P values of < 0.05 are shown in bold.

Genotyping call rate values of < 0.95 are shown in bold.

Supplementary Table 2. Linear regression models of associations between the MMSE scores and 35 tag SNPs in four interleukin-12 related genes including the *IL12A*, *IL12B*, *IL12RB1*, and *IL12RB2* genes.

**Gene**	**CHR**	**SNP**	**A1**	**A2**	**P (dominant)**	**P (recessive)**
IL12A	3	rs67717498	C	T	0.2749	0.7116
		rs9864047	C	G	0.3081	0.3083
		rs6762955	A	T	0.6204	0.8960
		rs77444103	T	C	0.5089	0.7454
		rs9840016	C	T	0.9975	0.4018
		rs116910715	A	G	0.3012	**0.0031**
		rs6795437	C	T	0.9019	0.0731
		rs78902931	G	A	0.7782	**0.0388**
		rs7631387	G	A	0.1475	0.6133
		rs480350	G	A	0.3322	0.8794
		rs117988966	A	G	0.4264	0.1254
		rs485789	T	G	0.1641	0.9835
		rs79458760	A	C	0.3532	0.9987
		rs67872958	C	T	0.7945	0.4643
		rs9880646	G	A	0.4764	0.9838
		rs78569420	T	G	0.6042	**0.0093**
IL12B	5	rs2853697	G	T	0.2960	0.3023
		rs3181216	T	A	0.7893	0.7807
		rs1003199	T	C	0.7502	0.0701
		rs730691	T	C	**0.0472**	0.8141
IL12RB1	19	rs445521	G	T	0.5320	0.7688
		rs383483	G	A	0.4138	0.4997
		rs7250425	C	T	0.2391	0.7719
IL12RB2	1	rs7544381	T	C	0.3812	0.1670
		rs3790558	G	T	**6.87×10^{-4}**	0.1186

		rs4655538	T	C	**0.0454**	0.9679
		rs2201584	A	G	0.7924	0.0883
		rs1495964	A	G	0.1820	0.3211
		rs17129794	C	A	0.9010	0.2963
		rs6693065	A	G	0.1662	0.6538
		rs114967702	G	A	0.5421	0.5514
		rs3790569	C	T	0.3377	0.4489
		rs79411372	T	C	0.4203	0.2196
		rs75699623	A	G	**0.0308**	0.8652
		rs1874396	G	T	**0.0425**	0.9891

A1 = minor allele, A2 = major allele, Chr = chromosome, MMSE = Mini-Mental State Examination.

P values of < 0.05 are shown in bold.

Analysis was obtained after adjustment for covariates including age, gender, and education.

Supplementary Table 3. Linear regression models of associations between the MMSE scores and 2 SNPs within the *IL12A* and *IL12B* genes.

						Dominant model			Recessive model		
**Gene**	**CHR**	**SNP**	**A1**	**A2**	**MAF**	**BETA**	**SE**	**P**	**BETA**	**SE**	**P**
IL12A	3	rs568408	A	G	0.117	0.15	0.11	0.1625	-0.07	0.42	0.8669
IL12B	5	rs3212227	A	C	0.442	-0.13	0.09	0.1800	-0.10	0.11	0.3621

A1 = minor allele, A2 = major allele, BETA = Beta coefficients, Chr = chromosome, HWE = Hardy–Weinberg equilibrium, MAF = minor allele frequency, MMSE = Mini-Mental State Examination, SE = standard error.

Analysis was obtained after adjustment for covariates including age, gender, and education.

Supplementary Table S4. MAF in various ethnic populations for 8 SNPs identified in our study.

Gene	CHR	SNP	A1	A2	MAF				
					**Taiwanese** [a]	**European** [b]	**East Asian** [b]	**African Americans**[b]	**South Asian** [b]
IL12A	3	rs116910715	A	G	0.065	0.000	0.057	0.000	0.000
		rs78902931	G	A	0.128	0.001	0.126	0.000	0.01
		rs78569420	T	G	0.083	0.000	0.103	0.000	0.000
IL12B	5	rs730691	T	C	0.440	0.377	0.504	0.654	0.47
IL12RB2	1	rs3790558	G	T	0.468	0.437	0.473	0.865	0.48
		rs4655538	T	C	0.186	0.023	0.163	0.269	0.09
		rs75699623	A	G	0.069	0.000	0.068	0.000	0.000
		rs1874396	G	T	0.227	0.025	0.200	0.318	0.09

A1 = minor allele, A2 = major allele, Chr = chromosome, MAF = minor allele frequency.

[a] Data from the present study.

[b] Data from the 1000 Genomes Project (https://www.ncbi.nlm.nih.gov/snp/).
